# The Tankyrase Inhibitor OM-153 Demonstrates Antitumor Efficacy and a Therapeutic Window in Mouse Models

**DOI:** 10.1158/2767-9764.CRC-22-0027

**Published:** 2022-04-20

**Authors:** Shoshy A. Brinch, Enya Amundsen-Isaksen, Sandra Espada, Clara Hammarström, Aleksandra Aizenshtadt, Petter A. Olsen, Lone Holmen, Merete Høyem, Hanne Scholz, Gunnveig Grødeland, Sven T. Sowa, Albert Galera-Prat, Lari Lehtiö, Ilonka A.T.M. Meerts, Ruben G.G. Leenders, Anita Wegert, Stefan Krauss, Jo Waaler

**Affiliations:** 1 Department of Immunology and Transfusion Medicine, Oslo University Hospital, Rikshospitalet, Oslo, Norway.; 2 Hybrid Technology Hub–Centre of Excellence, Institute of Basic Medical Sciences, Faculty of Medicine, University of Oslo, Oslo, Norway.; 3 Department of Pathology, Oslo University Hospital, Rikshospitalet, Oslo, Norway.; 4 Department of Transplant Medicine and Institute for Surgical Research, Oslo University Hospital, Rikshospitalet, Oslo, Norway.; 5 Institute of Clinical Medicine, Faculty of Medicine, University of Oslo, Oslo, Norway.; 6 Faculty of Biochemistry and Molecular Medicine, Biocenter Oulu, University of Oulu, Oulu, Finland.; 7 Symeres, Nijmegen, the Netherlands.

## Abstract

The catalytic enzymes tankyrase 1 and 2 (TNKS1/2) alter protein turnover by poly-ADP-ribosylating target proteins, which earmark them for degradation by the ubiquitin–proteasomal system. Prominent targets of the catalytic activity of TNKS1/2 include AXIN proteins, resulting in TNKS1/2 being attractive biotargets for addressing of oncogenic WNT/β-catenin signaling. Although several potent small molecules have been developed to inhibit TNKS1/2, there are currently no TNKS1/2 inhibitors available in clinical practice. The development of tankyrase inhibitors has mainly been disadvantaged by concerns over biotarget-dependent intestinal toxicity and a deficient therapeutic window. Here we show that the novel, potent, and selective 1,2,4-triazole–based TNKS1/2 inhibitor OM-153 reduces WNT/β-catenin signaling and tumor progression in COLO 320DM colon carcinoma xenografts upon oral administration of 0.33–10 mg/kg twice daily. In addition, OM-153 potentiates anti–programmed cell death protein 1 (anti–PD-1) immune checkpoint inhibition and antitumor effect in a B16-F10 mouse melanoma model. A 28-day repeated dose mouse toxicity study documents body weight loss, intestinal damage, and tubular damage in the kidney after oral–twice daily administration of 100 mg/kg. In contrast, mice treated oral–twice daily with 10 mg/kg show an intact intestinal architecture and no atypical histopathologic changes in other organs. In addition, clinical biochemistry and hematologic analyses do not identify changes indicating substantial toxicity. The results demonstrate OM-153–mediated antitumor effects and a therapeutic window in a colon carcinoma mouse model ranging from 0.33 to at least 10 mg/kg, and provide a framework for using OM-153 for further preclinical evaluations.

**Significance:**

This study uncovers the effectiveness and therapeutic window for a novel tankyrase inhibitor in mouse tumor models.

## Introduction

The ADP-ribosyltransferases family members tankyrase 1 and 2 (TNKS1/2) are protein-modifying enzymes at the crossroad of multiple cellular pathways (1–7). Using the redox metabolite NAD^+^, TNKS1/2 catalyze a posttranslational modification termed poly(ADP-ribosyl)ation which serves as a recognition signal for E3 ligase–mediated polyubiquitination followed by proteasomal degradation (1, 8–10). TNKS1/2 poly(ADP-ribosyl)ate several target proteins (1–7, 11–13), of which the proteins AXIN1, AXIN2 (AXIN1/2), and angiomotin (AMOT), angiomotin like 1 and 2 (AMOTL1/2) have received the largest attention as they are central in regulating the activity of the cellular wingless-type mammary tumor virus integration site (WNT)/β-catenin and Hippo signaling pathways, respectively (14, 15). TNKS1/2 inhibitor (TNKSi) treatment leads to stabilization of AXIN1/2, and hence stabilized β-catenin degradasomes, which in turn enhances degradation of the transcriptional regulator β-catenin resulting in inhibition of WNT/β-catenin signaling (14, 16, 17). In Hippo signaling, TNKSi-mediated AMOT protein stabilization facilitates changes in the subcellular location of the transcription cofactors yes-associated protein 1 YAP (YAP) and WW domain containing transcription regulator 1 (WWTR1/TAZ), leading to reduced YAP signaling (7, 15, 18). The WNT/β-catenin and Hippo signaling pathways are involved in a multitude of disease conditions, including tumorigenesis and tumor immune evasion (19–21). Hence, significant efforts have been made to develop selective TNKS1/2 inhibitors (14, 22–29). In particular, inhibitors based on the 1,2,4-triazole scaffold, such as JW74, OD336, G007-LK, OM-1700, and OM-153, target the adenosine binding pocket of the TNKS1/2 catalytic domains with high selectivity, and are therefore able to display selectivity over other PARP enzymes (22, 27, 30–32).

Clinical tankyrase inhibitor development has so far been hampered by concerns about target-specific and signaling pathway–specific side effects, in particular intestinal toxicity (5, 33, 34). Despite this, the tankyrase inhibitors E7449 and STP1002 have entered clinical trials in the cancer arena (35–38). This supports the potential of TNKS1/2 inhibitors and justifies the continued development of drugs directed toward TNKS1/2 inhibition. Recently, we developed the small-molecule 1,2,4-triazole-based TNKSi OM-153 through iterative design–make–test cycles possessing favorable drug properties. These properties include efficacy, off-target liabilities, absorption/distribution/metabolism/excretion (ADME) parameters, and pharmacokinetic profile in mice (32). Here, we evaluate the biological properties and potential toxicity issues of OM-153 in detail. First, we tested the efficacy of OM-153 in a panel of tumor cell lines showing low-nanomolar range biotarget engagement and tumor cell growth inhibition. Next, we carried out a standardized dose-escalating experiment using the COLO 320DM colon carcinoma xenograft model, showing significant multidose tumor growth inhibition, and also in an isogenic B16-F10 mouse melanoma model. Subsequently, the oral single-dose maximum tolerated dose (MTD) for OM-153 was established. The MTD was followed by a two-dose (10 mg/kg and 100 mg/kg), oral and twice daily 28-day repeated dose mouse toxicity evaluation, including multiorgan histopathology, biochemistry, and hematology in mice. With the applied methodology used in mice, we show no significant adverse toxicity in mice dosed oral–twice daily with 10 mg/kg, and anti–colon carcinoma efficacy when dosed oral–twice daily at 0.33–10 mg/kg. Hence, in contrast to the earlier studies (33, 34), our data propose the existence of a therapeutic window for OM-153 ranging from 0.33 to at least 10 mg/kg in a mouse colon carcinoma model.

## Materials and Methods

### Cell Culture and *In Vitro* Experiments

The cell lines where obtained from ATCC or the Japanese Collection of Research Bioresources (JCRB) in 2014 and culturing was performed as described previously (7). The cell cultures were kept below 20 passages (∼10 weeks) and routinely monitored (upon thawing and monthly) for *Mycoplasma* infections with MycoAlert Mycoplasma Detection Kit (Lonza). All cell lines were authenticated by short tandem repeat profiling to confirm their identity (Eurofins). TNKS1/2 and PARP1 biochemical assays (27, 32), luciferase-based WNT/β-catenin signaling pathway reporter assay (22, 27, 32), anti-proliferative assays, and NCI-60 tumor cell line panel screen (7), immunoblot analyses (7, 32, 39), RNA isolation, and real-time qRT-PCR (7, 27, 39, 40) were performed as previously described. Structured illumination microscopy (SIM) was performed as previously described (7, 39) using the following additional primary antibody: AXIN1 (2087, RRID: AB_2274550, Cell Signaling Technology) and displayed as maximum intensity projections rendered from 20 Z planes spanning 3.68 μm in total.

### Animal Experiments

The pharmacokinetic analyses were performed according to the standard protocols of Medicilon as previously described (22, 27, 32). Three CD-1 mice (999M-018, Sino-British SIPPR/BK Lab Animal) were treated by intravenous (i.v) (0.4 or 2 mg/kg) or oral (10 or 100 mg/kg) administrations of OM-153 (Symeres), once or twice daily (dosing at experiment start and after 6 hours), using 5% DMSO (D2650, Sigma-Aldrich), 50% PEG400 (06855, Sigma-Aldrich), and 45% saline as vehicle. The same vehicle and oral–twice daily treatment regime were used for all other experiments. Samples were collected after 15 and 30 minutes plus 1, 2, 4, and 6 hours for group 1, and after 6.5, 8, 10, and 24 hours for group 2.

The experiment using COLO 320DM (CCL-220, RRID: CVCL_0219, ATCC) xenografts was carried out as previously described (40) at Reaction Biology. On day 3 after subcutanous (s.c.) tumor challenge in CB17-SCID mice (CRL: 561, RRID: IMSR_CRL: 561, Charles River), tumor-bearing animals (mean tumor volume: 12 mm^3^) were randomized into six groups (all *n* = 10). The day after, the animals were treated oral–twice daily with 10, 3.3, 1, 0.33, or 0.1 mg/kg OM-153 or vehicle until day 37. Eight mice, evenly distributed between the treatment groups, were euthanized for ethical reasons before experiment termination, due to skin ulcerations in the tumor area (6 mice) or body weight loss (>20%, two mice). Next, the mice were sacrificed, approximately 4 hours after first dose of the day. Protein and RNA extracts were prepared from four moderately sized tumors within each treatment group. Mass spectrometry analyses of OM-153 in plasma and tumors were performed according to the standard protocols of Pharmacelsus.

The experiment using B16-F10 tumors was carried out as previously described (39) at Reaction Biology. On day six after tumor challenge (s.c.) in C57BL/6N mice (CRL: 493, RRID: IMSR_CRL: 493, Charles River), tumor-bearing animals (mean tumor volume: 31 mm^3^) were randomized into six groups (all *n* = 15). On the same day, the animals were treated oral–twice daily with vehicle, anti-programmed cell death 1 (anti–PD-1; 10 mg/kg i.p. on day 6, 9, and 12, BE0033–2, RRID: AB_1107747, Bio X Cell), 10 mg/kg OM-153, or combined treatment with anti–PD-1 and 10, 1, and 0.1 mg/kg OM-153 until day 20. Fifteen mice, distributed between the treatment groups, were euthanized for ethical reasons before experiment termination, due to skin ulcerations in the tumor area. Next, the mice were sacrificed, approximately 8 hours after last dosing. Protein and RNA extracts were prepared from 8 tumors within each treatment group.

The dose escalation experiment was performed in single male CD-1 mice (CRL: 022, RRID: IMSR_CRL: 022, Charles River) at BSL BIOSERVICE/Eurofins using their standard protocol. The animals were treated oral–twice daily with escalating doses of 500, 1,000, 1,500, and 2,000 mg/kg OM-153, and observed for 48 hours for clinical signs (7 days for the verification animal, 2,000 mg/kg). Body weight was recorded daily.

The 28-day oral repeated dose toxicity study was performed at Reaction Biology (10 mg/kg) and at Oslo University Hospital (100 mg/kg) using male CD-1 (CRL: 022, RRID: IMSR_CRL: 022, Charles River) and C57BL/6J (JAX: 000664, RRID: IMSR_JAX: 000664, Jackson) mice, respectively. The mice were treated oral–twice daily with 10 (*n* = 7) or 100 (*n* = 4) mg/kg OM-153 or vehicle control (both experiments, *n* = 4) until day 28, or study termination (for 100 mg/kg). Body weight and food consumption were measured thrice weekly. For the experiment using 10 mg/kg oral–twice daily dosing, blood was collected at the end of the experiment and hematologic and clinical biochemistry analyses were performed at IDEXX according to their standard protocols. Duodenum, jejunum, ileum, colon–rectum, lymph nodes, pancreas, heart, kidney, liver, spleen, lung, prostate, and testis were collected, formalin-fixed (10%), paraffin-embedded, stained with hematoxylin and eosin (H&E), and imaged as previously described (39). Staining of ileums using immunofluorescence was performed as previously described (29, 39). Donkey anti-rabbit IgG cyanine Cy3 was used as secondary antibody [(550 nm), 1:500, 711165152, RRID: AB_2307443, Jackson ImmunoResearch] for 60 minutes at 37°C. DAPI nuclear dye [(340 nm), 1 μg/μL, D9542, Sigma Aldrich] was added to the final washing solution. RNAscope multiplex fluorescent reagent kit v2 detection (323100, Advanced Cell Diagnostics) was used to detect expression of mouse leucine-rich repeat-containing G-coupled receptor 5 (*Lgr5*) mRNA (312171, Advanced Cell Diagnostics) according to the manufacturer's instructions. Imaging was performed using confocal microscopy as described previously (39). All animal experiments were approved by local animal experiment authorities (ethics committee of the Chinese Association for Laboratory Animal Sciences, German Centre for the Protection of Laboratory Animals, and Norwegian Food Safety Authority), and were carried out in compliance with FELASA guidelines and recommendations.

### Quantification and Statistical Analysis

Statistical analyses were performed as previously described using *t* tests and Mann–Whitney rank sum tests (7).

### Data Availability Statement

The data generated in this study are available within the article and its Supplementary Data files. Materials generated in this study can be made available upon request to the corresponding author.

## Results

### OM-153 Specifically Inhibits TNKS1/2, WNT/β-Catenin Signaling, and Cell Growth of an *APC-*Mutated Colon Carcinoma Cell Line

**FIGURE 1:**
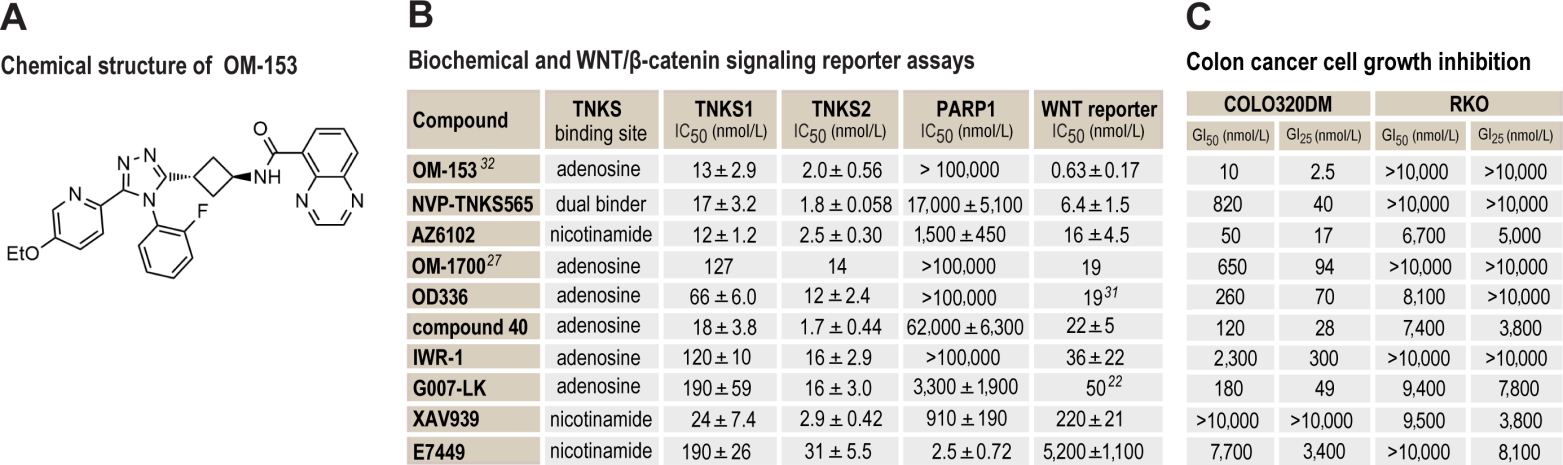
OM-153 specifically inhibits TNKS1/2, WNT/β-catenin signaling and cell growth of an *APC-*mutated colon cancer cell line. **A,** Chemical structure of OM-153. **B,** IC_50_ values for inhibition of TNKS1, TNKS2, and PARP1 (biochemical assays) and luciferase-based WNT/β-catenin signaling reporter assay in HEK293 cells (WNT reporter). The table is sorted in ascending order according to the WNT reporter IC_50_ values. Mean ± SD values from a minimum of three independent experiments are shown. For **B** and **C**, numbers in superscript indicate data from reference 22, 27, 31, and 32. As indicated, compounds inhibit TNKS1/2 by binding to the catalytic domain, either in the nicotinamide pocket or in adenosine pocket, or in both (dual binder). **C,** MTS colorimetric cell growth assay for various concentrations of tankyrase inhibitors in treated COLO 320DM and RKO colon cancer cells. GI_50_ and GI_25_ values were calculated relative to controls (100%, 0.01% DMSO) and experiment time 0 values (*t*_0_, set to 0%) after 5 days of cultivation. Data from one representative experiment of more than three repeated assays, each with six replicates, are shown.

OM-153 was recently developed and shows an overall favorable drug property profile (ref. 32; Fig. 1A). To evaluate potency and specificity, OM-153 was tested in biochemical TNKS1/2 and PARP1 assays, and in a luciferase-based WNT/β-catenin signaling reporter assay. OM-153 was compared with a selected TNKSi panel consisting of NVP-TNKS565 (24), AZ6102 (25), compound 40 (23), IWR-1 (28), XAV939 (14), E7449 (35), and previously identified 1,2,4-triazole-based TNKS1/2 inhibitors (Supplementary Fig. S1; refs. 22, 27, 31). OM-153 that binds to the adenosine site of the TNKS1/2 catalytic domains (32), decreased the activities of TNKS1 and TNKS2 with IC_50_ values (concentrations resulting in 50% inhibition) of 13 and 2.0 nmol/L, respectively, approaching the technical limit of the assay due to required protein concentration (Fig. 1B; Supplementary Fig. S2A and S2B). The IC_50_ value for PARP1 inhibition was >100,000 nmol/L (Fig. 1B; Supplementary Fig. S2C). In contrast, compounds that bind to the nicotinamide pocket also inhibited PARP1 activity (Fig. 1B; Supplementary Fig. S2C). OM-153 inhibited luciferase-based WNT/β-catenin signaling reporter activity with an IC_50_ value of 0.63 nmol/L (Fig. 1B; Supplementary Fig. S2D).

Next, in comparison with the selected TNKSi panel, the potential of OM-153 as an antiproliferative agent was tested in a cell growth assay in the adenomatous polyposis coli (*APC*)*-*mutated, WNT/β-catenin signaling–dependent, and TNKSi-sensitive colon carcinoma cell line COLO 320DM (7, 32, 33, 40). To evaluate the specificity of the cell growth inhibition, the *APC–*wild-type, WNT/β-catenin signaling–independent and TNKSi-insensitive colon carcinoma cell line RKO was used as a control (7, 29, 32). Treatment with OM-153 decreased cell growth in COLO 320DM cells with a GI_50_ value of 10 nmol/L and a GI_25_ value of 2.5 nmol/L (concentrations resulting in 50% and 25% growth inhibition, respectively), while cell growth in RKO cells was insubstantially affected by the treatment (GI_50_ and GI_25_ values >10,000 nmol/L; Fig. 1C; Supplementary Fig. S3).

In conclusion, in addition to our recent report (32), the results demonstrate that OM-153 is a highly potent and specific inhibitor of TNKS1/2, WNT/β-catenin signaling and cell growth in the APC-mutated colon carcinoma cell line COLO 320DM.

### OM-153 Inhibits WNT/β-Catenin, YAP, and MYC Signaling and Shows an Antiproliferative Effect in Human Cancer Cell Lines

**FIGURE 2:**
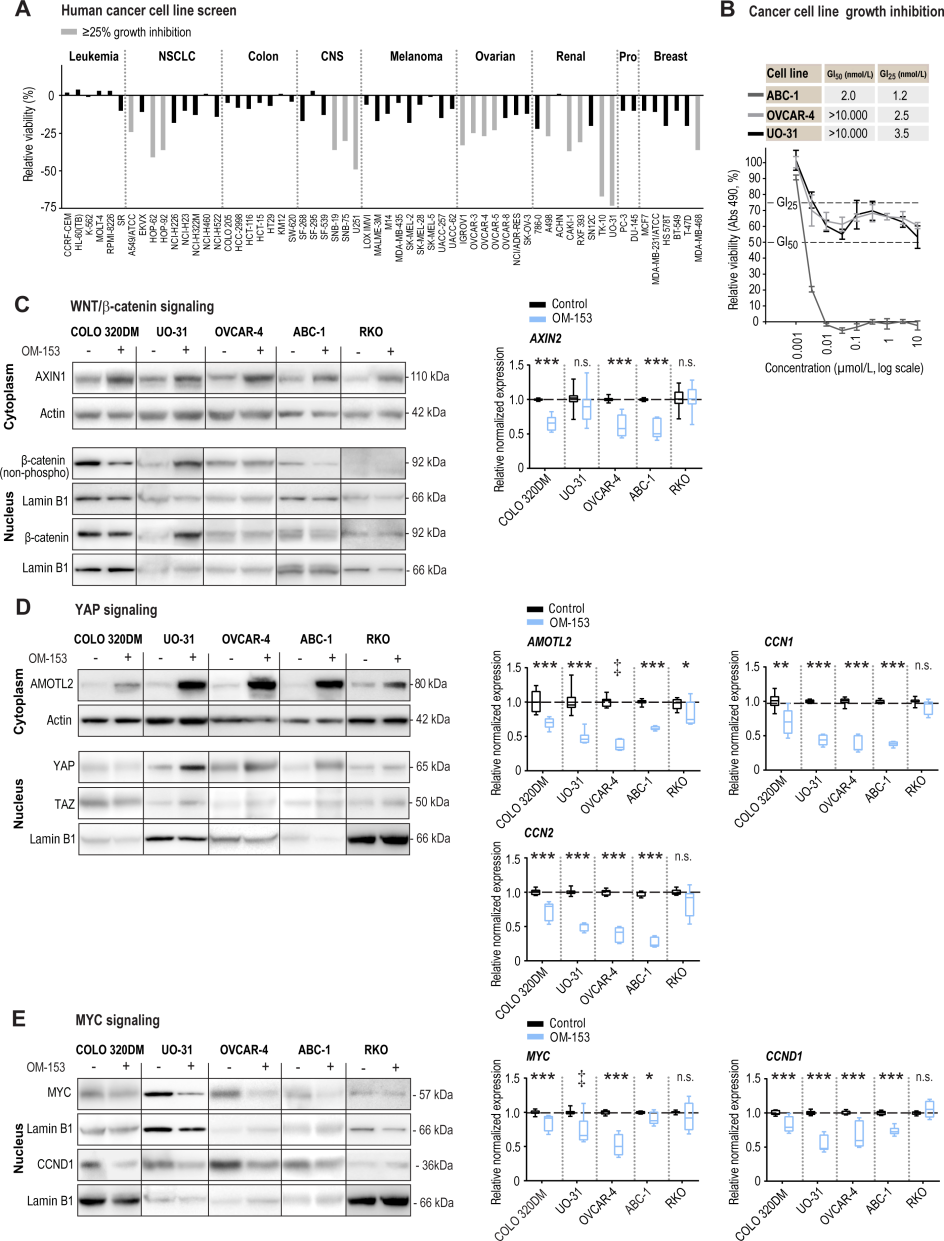
OM-153 inhibits WNT/β-catenin, YAP, and MYC signaling and shows an antiproliferative effect in human cancer cell lines. **A,** NCI-60 human cancer cell line proliferation/viability screen using OM-153 (10 nmol/L) for 48 hours and ≥ 25% relative growth inhibition is highlighted by gray bars (control, 0.01% DMSO = 0%). Prostate (Pro) and central nervous system (CNS). **B,** MTS colorimetric cell growth assay for various concentrations of OM-153 in treated ABC-1, OVCAR-4, and UO-31 cells. GI_50_ and GI_25_ values (nmol/L) were calculated relative to control (100%, 0.01% DMSO) and experiment time 0 values (Abs 490 t_0_, set to 0%) after 5 days of cultivation. Mean ± SD values for data from one representative experiment of more than three repeated assays, each with six replicates, are shown. **C,** Evaluation of WNT/β-catenin signaling. Left, immunoblots of cytoplasmic AXIN1 and nuclear active form of β-catenin [non-phospho (Ser33/37/Thr41)] and total β-catenin. Lamin B1 loading controls are duplicated for OVCAR-4 immunoblots. Right, real-time RT-qPCR analysis of the WNT/β-catenin signaling target gene *AXIN2*. For **C**–**E**, after 24 hours of treatment with OM-153 (10 nmol/L) or controls (0.0001% DMSO) in COLO 320DM, UO-31, OVCAR-4, ABC-1, and RKO. Immunoblots show representative data from two or more independent experiments. Actin (cytoplasm) and lamin B1 (nucleus) were used as loading controls. Boxplots show median, first, and third quartiles and maximum and minimum whiskers for combined data from three independent experiments with three replicates each. Stippled lines (black) depict control mean values = 1. Two-tailed *t* tests: ***, *P* < 0.001; **, *P* < 0.01; and *, *P* < 0.05. Mann–Whitney rank sum tests: ‡, *P* < 0.01. ns., not significant. **D,** Evaluation of YAP signaling. Left, immunoblots of cytoplasmic AMOTL2 (top) and nuclear YAP and TAZ (bottom). Right, real-time RT-qPCR analysis of the YAP signaling target genes *AMOTL2*, *CCN1*, and *CNN2*. For **D** and **E**, lamin B1 loading controls are duplicated for RKO immunoblots (YAP/TAZ and CCND1). **E,** Evaluation of MYC signaling. Left, immunoblots of nuclear MYC and CCND1. Lamin B1 loading controls are duplicated for ABC-1 immunoblots. Right, real-time RT-qPCR analysis of the MYC signaling target genes *MYC* and *CCND1*.

Recently, we showed that TNKS1/2 inhibition could attenuate cell growth in a wide range of cancer types *in vitro* (7). To evaluate cancer cell line growth inhibition by OM-153, the NCI-60 tumor cell line panel was screened. Of the 60 tested cancer cell lines, 16 cell lines showed >25% relative growth inhibition upon treatment with 10 nmol/L OM-153 (Fig. 2A; Supplementary Fig. S4). These cell lines originated from lung, brain, ovary, and kidney, and included the previously identified TNKSi-sensitive cell lines OVCAR-4 (ovarian cancer; ref. 7) and UO-31 (renal cancer; ref. 7). These two cell lines along with the highly TNKSi-sensitive cell line ABC-1 (non–small cell lung cancer; ref. 7), were treated with various doses of OM-153 for five days. Treatment with OM-153 showed a cytotoxic effect in ABC-1 cells with a GI_50_ value of 2.0 nmol/L and a GI_25_ value of 1.5 nmol/L (Fig. 2B). In OVCAR-4 and UO-31 cells, cytostatic effects were observed with GI_25_ values of 2.5 and 3.5 nmol/L, respectively (Fig. 2B). In conclusion, low nanomolar concentrations of OM-153 can reduce cell growth in a subset of cancer cell lines *in vitro*.

**FIGURE 3:**
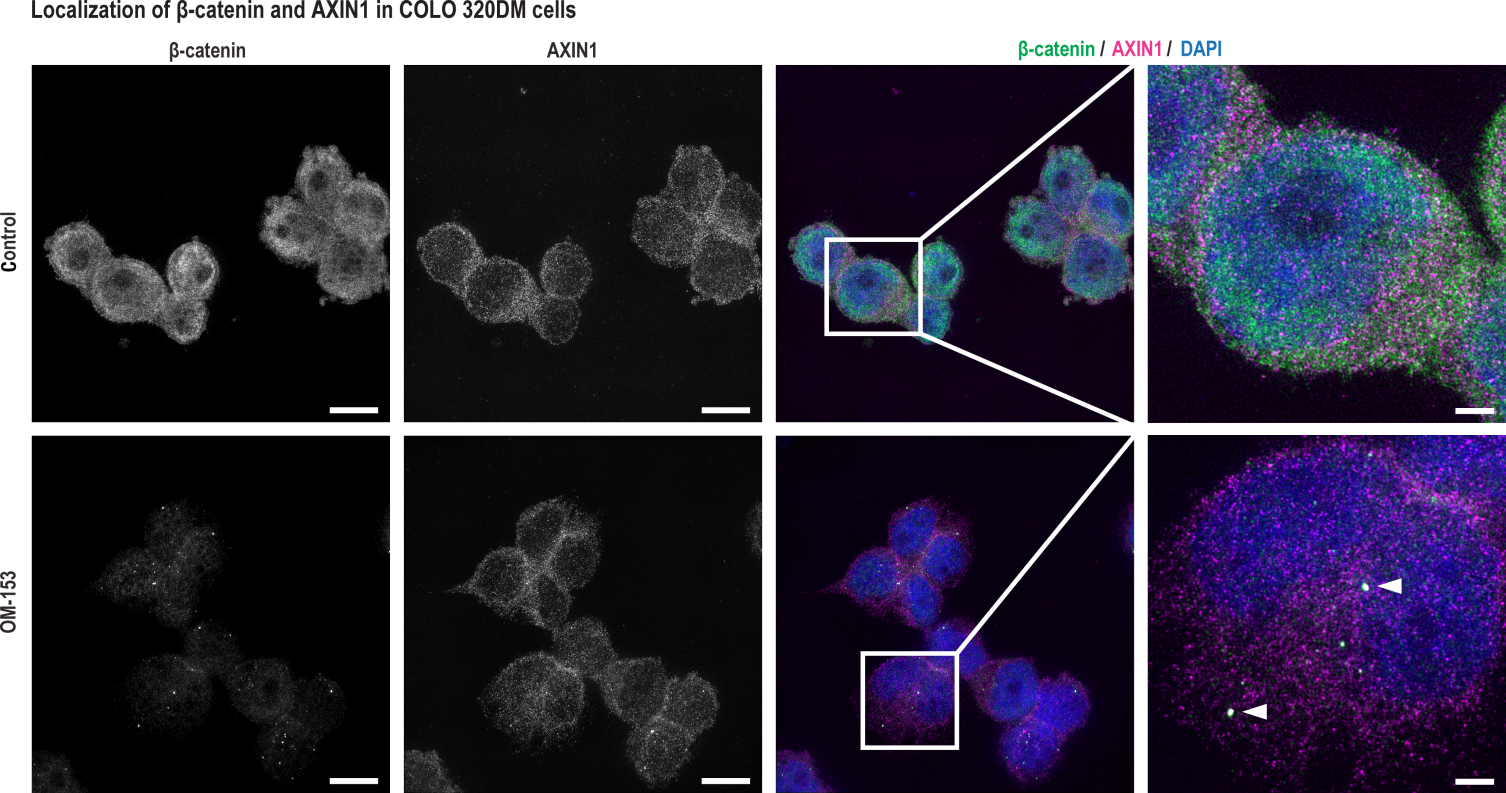
OM-153 induces formation of β-catenin and AXIN1-containing puncta and reduces nuclear β-catenin in COLO 320DM cells. Representative SIM images of β-catenin and AXIN1 after 24 hours of treatment with OM-153 (10 nmol/L) or controls (0.0001% DMSO) in COLO 320DM. Left, single-channel images in grayscale. Right, merged images of β-catenin (green), AXIN1 (magenta), and nuclear DAPI staining (blue). Arrows highlight colocalization of β-catenin and AXIN1. Scale bars, 10 μm and 2 μm (magnification).

Recently, we showed that TNKS1/2 inhibition cell-type dependently can block WNT/β-catenin and/or YAP signaling, and consequently MYC proto-oncogene bHLH transcription factor (MYC) signaling, resulting in attenuated cancer cell growth (7). Hence, immunoblot and real-time qRT-PCR analyses were conducted to examine the effect of treatment with 10 nmol/L OM-153 against these signaling pathways in a panel of cells consisting of the TNKSi-sensitive cell lines COLO 320DM (Fig. 1C; Supplementary Fig. S3), UO-31, OVCAR-4, and ABC-1 (7). The TNKSi-insensitive colon carcinoma cell line RKO was included as a control (Fig. 1C; Supplementary Fig. S3; refs. 7, 29). First, the effects of OM-153 treatment on WNT/β-catenin signaling components were tested. OM-153 stabilized the β-catenin degradasome–forming protein AXIN1 in all cell lines (Fig. 2C). A reduction of the transcriptionally active form of β-catenin in the nuclei was only documented in COLO 320DM and ABC-1 cells (Fig. 2C; Supplementary Fig. S5A). To investigate OM-153–mediated control of WNT/β-catenin signaling, real-time qRT-PCR analysis of the cell type–independent and universal target gene *AXIN2* was performed. The analysis showed significantly reduced transcription of *AXIN2*, demonstrating attenuation of WNT/β-catenin signaling in COLO 320DM, OVCAR-4 and ABC-1 cells (Fig. 2C). Moreover, SIM microscopy revealed the formation of cytoplasmic puncta containing AXIN1 and colocalizing β-catenin, indicating accumulation of β-catenin degradasomes (7, 17) in COLO 320DM cells (Fig. 3).

Next, the effect of OM-153 treatment on YAP signaling was examined. Consistent with earlier research (7), an immunoblot analysis showed cytoplasmic stabilization of the TNKS1/2 target protein AMOTL2 in all cell lines upon OM-153 treatment, while nuclear YAP accumulation was only seen in UO-31, OVCAR-4, and ABC-1 cells (Fig. 2D; Supplementary Fig. S5B). Moreover, the real-time qRT-PCR analysis showed reduced transcription of the YAP signaling target genes *AMOTL2*, cellular communication network factor 1 and 2 (*CCN1* and *CCN2*) in all TNKSi-sensitive cell lines (Fig. 2D).

Finally, the effect of OM-153 treatment on MYC expression was assessed. Immunoblot and real-time qRT-PCR analyses showed that MYC and cyclin D1 (CCND1) protein, as well as transcription of *MYC* and the MYC signaling target gene *CCND1*, were decreased in all TNKSi-sensitive cell lines (Fig. 2E; Supplementary Fig. S5C).

In conclusion, OM-153 functions as a potent cell type–dependent inhibitor of WNT/β-catenin, YAP and MYC signaling and can reduce cell growth in a subset of human cancer cell lines in cell culture.

### OM-153 Inhibits WNT/β-Catenin Signaling and Shows Antitumor Effect in a Human Colon Carcinoma Xenograft Model

**FIGURE 4:**
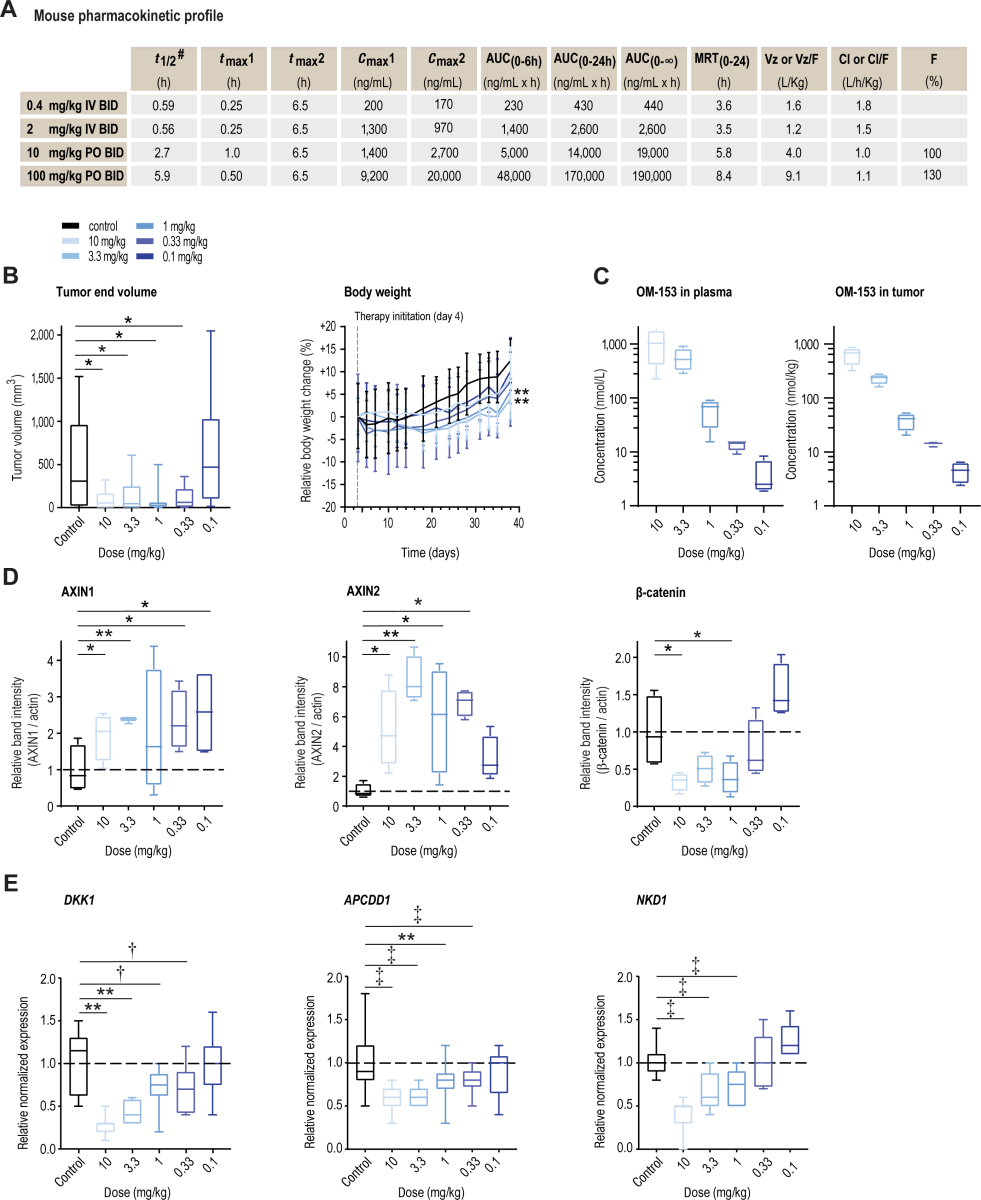
OM-153 inhibits WNT/β-catenin signaling and shows antitumor effect in a human colon carcinoma xenograft model. **A,** Mouse pharmacokinetic profile upon intravenous (i.v., 0.4 or 2 mg/kg) or oral (p.o., 10 or 100 mg/kg) administration of OM-153 either once (group 1) or twice daily (BID, group 2) in CD-1 mice. Half-life (*t*_1/2_^#^, calculated by setting the terminal phase from 8 hours to 24 hours for oral dosing, and 6.5 hours to 24 hours for intravenous dosing), time to achieve first and second dose *C*_max_ (*t*_max_1 and 2), maximum concentration reached (*C*_max_1 and 2), AUC, mean residence time (MRT), volume of distribution (Vz or Vz/F), clearance (Cl or Cl/F), and fraction absorbed/bioavailability (F). **B,** Tumor end volume (mm^3^), left. Relative body weights, right. Mean body weight at experiment initiation is set at 0%. ** indicates statistically significant body weight reductions after treatment with 3.3 and 1 mg/kg OM-153. Mean ± SD values are shown. For **B**–**E**, from COLO 320DM-challenged (s.c.) CB17-SCID mice upon treatment with 10 (*n* = 8), 3.3 (*n* = 10), 1 (*n* = 9), 0.33 (*n* = 9), or 0.1 (*n* = 9) mg/kg OM-153 and vehicle control (*n* = 7) from day 4 through 37 (all oral–twice daily). Boxplots show median, first, and third quartiles and maximum and minimum whiskers. One-tailed *t* tests: **, *P* < 0.01; *, *P* < 0.05. One-tailed Mann–Whitney rank sum tests: ‡, *P* < 0.01; and †, *P* < 0.05. For **C**–**E**, *n* = 4 tumors, collected 4 hours post last dosing, were analyzed for each treatment group. For **D** and **E**, stippled lines depict control mean values = 1. **C,** Mass spectrometry analyses of OM-153 in plasma (nmol/L, left) and tumor (nmol/kg, right). **D,** Representative and quantified protein immunoblot ratios (protein vs. loading control) showing altered expression of AXIN1, AXIN2 and β-catenin (total). **E,** Real-time RT-qPCR analyses of WNT/β-catenin signaling target genes *DKK1*, *APCDD1*, and *NKD1*.

A mouse pharmacokinetic analysis was designed to evaluate drug exposure for the mouse efficacy and toxicity studies using oral administration of 10 or 100 mg/kg OM-153, dosed twice daily at the start of the experiment and after 6 hours. After the second dose of 10 mg/kg OM-153, a plasma concentration (*C*_max_2) of 2,700 ng/mL (5.3 μmol/L) was observed. Although OM-153 was below the detection threshold after 24 hours, these results suggest a satisfactory timeframe with an efficacious concentration *in vivo* relative to the COLO 320DM *in vitro* GI_50_ value (5.1 ng/mL = 10 nmol/L; Fig. 4A; Supplementary Fig S6A). No corrections for plasma- and tissue-protein binding were performed for the *in vitro* experiments. At a 100 mg/kg oral–twice daily dosing, a *C*_max_2 of 20,000 ng/mL (39.2 μmol/L) was observed, and 1,900 ng/mL (3.7 μmol/L) were still detected after 24 hours (Fig. 4A; Supplementary Fig. S6A).

To evaluate whether the *in vitro* anti-proliferative effect of OM-153 could be translated to an *in vivo* setting, COLO 320DM tumor-challenged CB17-SCID mice were treated oral–twice daily with five doses of OM-153. Doses in the range of 0.33–10 mg/kg resulted in 74%–85% tumor end volume inhibition when compared with the control. Importantly, no changes in body weights that would be suggestive for toxicity were observed among mice receiving 10 mg/kg oral–twice daily (Fig. 4B; Supplementary Fig. S6B–S6E).

Next, mass spectrometry analyses were performed at the experimental endpoint to measure the concentrations of OM-153 in plasma and tumors. The analyses revealed a dose-dependent detection of OM-153 above 10 nmol/L for doses in the range of 0.33–10 mg/kg in both plasma and tumors (Fig. 4C). To evaluate WNT/β-catenin signaling biomarkers tumors were analyzed by immunoblot and real-time qRT-PCR. OM-153 treatment stabilized AXIN1 and AXIN2 proteins in the tumor specimen, while β-catenin protein and the WNT/β-catenin signaling target genes dickkopf WNT signaling pathway inhibitor 1 (*DKK1*), APC downregulated 1 (*APCDD1*), and NKD inhibitor of WNT signaling pathway 1 (*NKD1*) were dose-dependently reduced (Fig. 4D and E; Supplementary Fig. S6E).

In summary, the results show that oral–twice daily dosing of 0.33–10 mg/kg OM-153 can attenuate WNT/β-catenin signaling, showing a pronounced antitumor efficacy in the COLO 320DM colon carcinoma mouse xenograft model.

### Combined OM-153 and Anti–PD-1 Treatment Confers Antitumor Effect in Mouse Melanoma

Recently, we reported that TNKS1/2 inhibition potentiated the effect of PD-1 antibody–based immunotherapy against murine B16-F10 melanoma tumors, an anti–PD-1–resistant model showing β-catenin–induced immune evasion (39). The study documented that the combined treatment effect was dependent on loss of β-catenin in the tumor cells and induction of a CD8^+^ T cell–mediated adaptive antitumor immune response (39).

**FIGURE 5:**
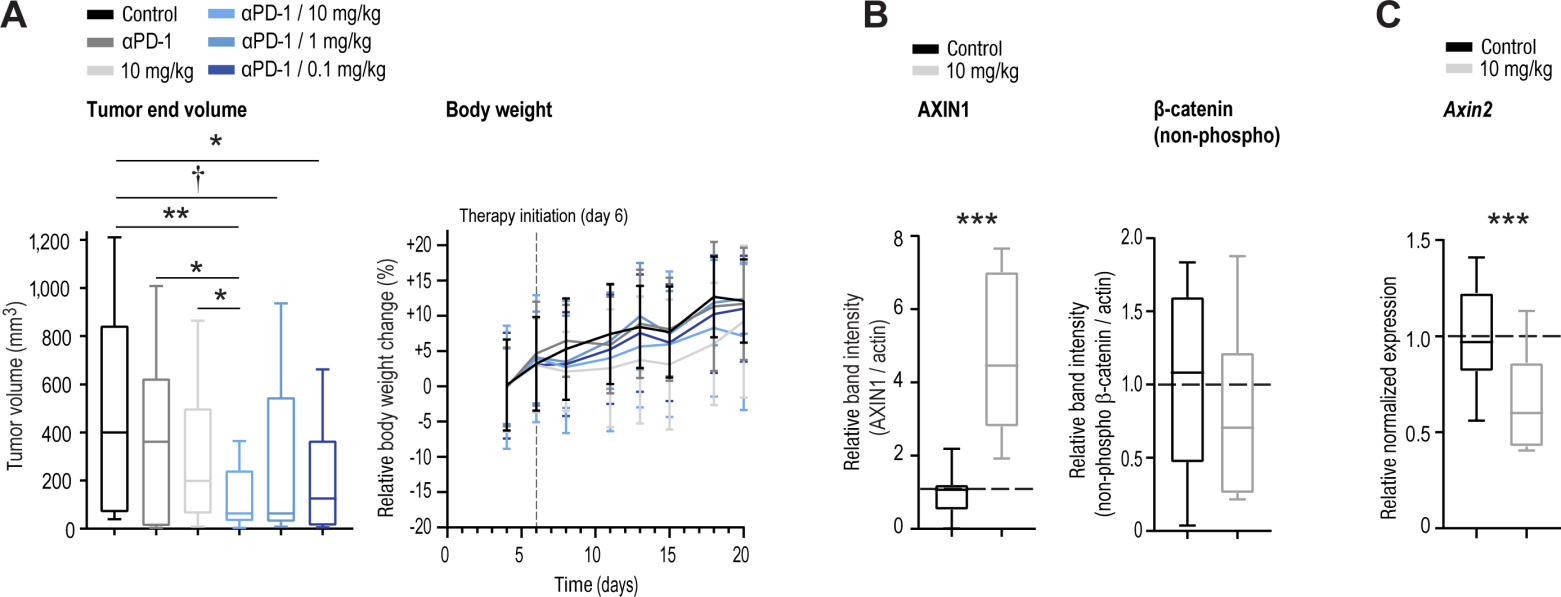
Combined OM-153 and anti–PD-1 treatment confers antitumor effect in mouse melanoma. **A,** Tumor end volume (mm^3^), left. Relative body weights, right. Mean body weight at experiment initiation is set at 0%. Mean ± SD values are shown. For **A–C**, from B16-F10-challenged (s.c.) C57BL/6N mice upon treatment from day 6 through 20. Vehicle control (*n* = 12), 10 mg/kg anti-(α)PD-1 dosed intraperitoneally on day 6, 9, and 12 (*n* = 13), 10 mg/kg OM-153 (*n* = 11), or combined treatment with αPD-1 and 10 (*n* = 13), 1 (*n* = 13), and 0.1 (*n* = 13) mg/kg OM-153. All OM-153 treatments, oral–twice daily. Boxplots show median, first and third quartiles and maximum and minimum whiskers. One-tailed *t* tests: ***, *P* < 0.001; **, *P* < 0.01; and *, *P* < 0.05. One-tailed Mann–Whitney rank sum test: †, *P* < 0.05. **B,** Representative and quantified protein immunoblot ratios (protein vs. loading control) showing altered expression of AXIN1 and the active form of β-catenin [non-phospho (Ser33/37/Thr41)]. For **B** and **C**, tumor analysis upon treatment with vehicle control and 10 mg/kg OM-153 (both *n* = 8), collected 8 hours post last dosing, and stippled lines depict control mean values = 1. **C,** Real-time RT-qPCR analyses of the WNT/β-catenin signaling target gene *Axin2*.

To examine the effect of combining OM-153 with anti-PD-1 treatment, B16-F10 tumors were established in immunocompetent C57BL/6N mice, followed by oral–twice daily treatment using various doses of OM-153, combined with three treatments of anti–PD-1 delivered three days apart. Similar to our previous report, OM-153 and anti–PD–1 monotherapy controls did not exert any significant antitumor effects (ref. 39; Fig. 5A; Supplementary Fig. S7A–S7C). However, when OM-153 was dosed in the range of 0.1–10 mg/kg in combination with anti–PD-1 treatment, 51%–65% tumor end volume inhibition was documented when compared with the vehicle control (Fig. 5A; Supplementary Fig. S7A–S7C). No alterations in body weights were observed in any of the treatment groups (Fig. 5A).

Tumors treated oral–twice daily with 10 mg/kg OM-153 exhibited significant stabilization of AXIN1 protein, but showed insignificant downregulation of β-catenin in an immunoblot analysis (Fig. 5B; Supplementary Fig. S7D). The real-time qRT-PCR analysis showed significantly decreased transcription of the target gene *Axin2*, indicating attenuated WNT/β-catenin signaling (Fig. 5C).

In summary, OM-153 can decrease WNT/β-catenin signaling in B16-F10 tumors and sensitize B16-F10 melanoma tumors to anti-PD-1 immunotherapy.

### Treatment with 10 mg/kg OM-153 Twice Daily Is Tolerated in Mice

TNKS1/2 inhibition has been associated with the induction of intestinal toxicity linked to biotarget and WNT/β-catenin signaling pathway-specific effects (33, 34). Hence, we performed a dose escalation experiment to evaluate acute toxicity and the MTD of OM-153 in mice. No clinical signs of systemic toxicity were observed in any animals after oral–twice daily dosing with 500, 1,000, 1,500, or 2,000 mg/kg of OM-153 (Supplementary Fig. S8A).

**FIGURE 6:**
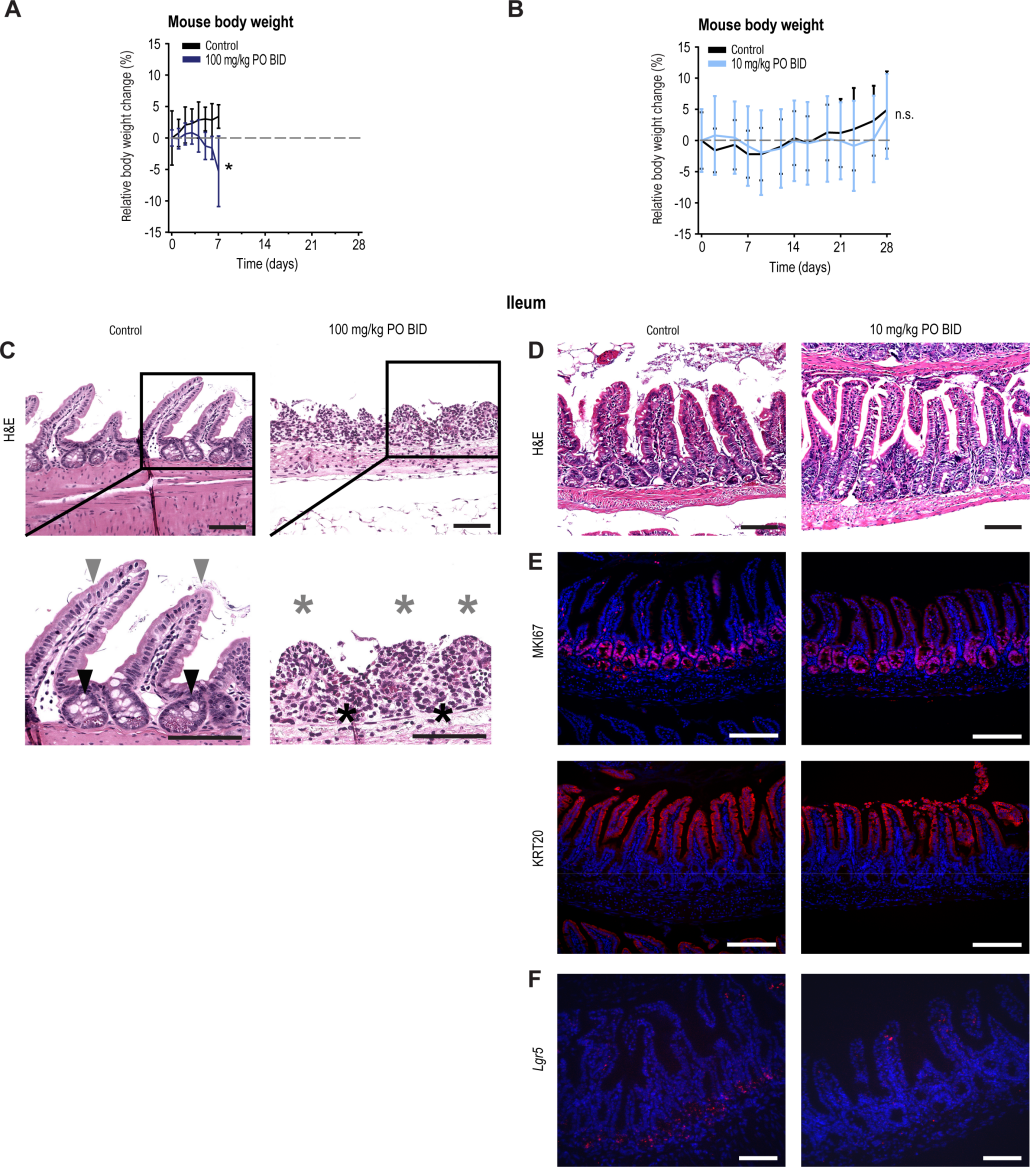
Oral–twice daily treatment with 10 mg/kg OM-153 does not induce intestinal toxicity in mice. **A,** Relative body weights upon 7 days of treatment with 100 mg/kg OM-153 (*n* = 4) and vehicle control (*n* = 4) in C57BL/6J mice. For **A** and **B**, mean body weight at experiment initiation is set at 0%. All treatments, oral–twice daily (p.o. BID). Means ± SDs are shown and two-tailed *t* test: *, *P* < 0.05. n.s., not significant. **B,** Relative body weights upon 28 days of treatment with 10 mg/kg OM-153 (*n* = 7) and vehicle control (*n* = 4) in CD-1 mice. **C,** Treated as described in **A** and stained with H&E. For zoom images, arrowheads highlight intact intestinal villi (gray) and crypts (black), while asterixes highlight loss of villi (gray) and crypts (black). For **C**–**F,** representative pictures, taken from multiple sections of ileums. For **C**–**E,** scale bars: 100 μm (original magnification × 200). **D,** Stained with H&E. For **D**–**F**, treated as described in **B**. **E,** MKI67 (magenta) and KRT20 (red) visualized using immunofluorescence. **F,***Lgr5* (magenta) visualized using RNAscope. Scale bars, 100 μm (original magnification ×100).

**FIGURE 7:**
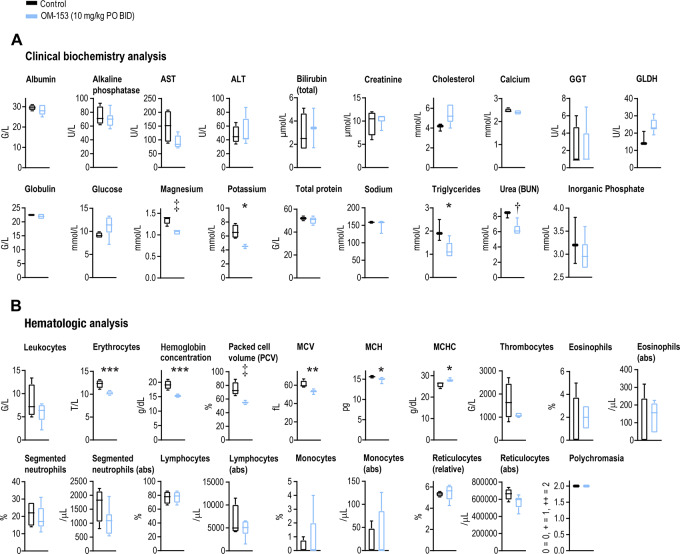
Clinical biochemistry and hematologic analyses of mice administered oral–twice daily dosing with 10 mg/kg OM-153 in a 28-day toxicity evaluation. **A,** Clinical biochemistry analysis. Aspartate aminotransferase (AST), alanine transaminase (ALT), gamma-glutamyl transferase (GGT), glutamate dehydrogenase (GLDH), blood urea nitrogen (BUN), grams/liter (g/L), units/liter (U/L), micromolar (μmol/L), and millimolar (mmol/L). For **A** and **B**, upon 28 days of treatment with 10 mg/kg OM-153 (*n* = 7) or vehicle control (*n* = 4), both ora–twice daily (p.o. BID) dosing in CD-1 mice. Boxplots show median, first, and third quartiles and maximum and minimum whiskers. Two-tailed *t* tests: ***, *P* < 0.001; **, *P* < 0.01; *, *P* < 0.05. Mann–Whitney rank sum tests: ‡, *P* < 0.01; †, *P* < 0.05. **B,** Hematologic analysis. Mean corpuscular volume (MCV), mean corpuscular hemoglobin (MCH), MCH concentration (MCHC), 10^9^ (giga)/liter (G/L), 10^12^ (tera)/liter (T/L), grams/deciliter (g/dL), femtoliter (fL), pictogram (pg), and per microliter (/μL). Basophils, band neutrophils, anisocytosis, or atypical cells were not detected.

To evaluate the long-term effects of OM-153 treatment, a 28-day oral repeated dose toxicity study was carried out using oral–twice daily dosing of 100 and 10 mg/kg. In mice dosed with 100 mg/kg, reduced activity and body weight loss in 3 of 4 mice were documented, and the mice were euthanized for ethical reasons on day 7 (Fig. 6A; Supplementary Fig. S8B). In contrast, in mice dosed with 10 mg/kg, no clinical signs, body weight loss, or reduced food consumption were observed (Fig. 6B; Supplementary Fig. S8C and S8D). Next, duodenum, jejunum and ileum, colon-rectum, lymph nodes, pancreas, heart, kidney, liver, spleen, lung, prostate and testis were collected, stained with H&E and the histopathology was evaluated. In the small intestines of 2 out of three 100 mg/kg treated mice, loss of villi height, width, crypt, and as well as surface epithelium, accompanied by inflammation, was documented (Fig. 6C; Supplementary Fig. S8E). In the same mice, signs of acute tubular damage in the kidney, presumably caused by intestinal damage, were observed (Supplementary Fig. S8E). No abnormal histopathologic changes in the other organs were detected (Supplementary Fig. S8E). On the contrary, the overall intestinal architecture was intact in the 10 mg/kg group (Fig. 6D). Persistent proliferation in the crypt compartments was indicated by visualization of the proliferation and intestinal stem cell (ISC)-like marker antigen identified by mAb Ki 67 (MKI67; Fig. 6E). Keratin 20 (KRT20) staining specified presence of terminally differentiated epithelial cells in the villus termini (Fig. 6E). Similar to previous observations (33, 41), expression of the ISC marker and WNT/β-catenin signaling target gene *Lgr5* was reduced (Fig. 6F). No atypical histopathologic changes in other organs were observed (Supplementary Fig. S8E). Blood was collected at the end of the experiment using 10 mg/kg dosing to perform clinical biochemistry and hematologic analyses. The clinical biochemistry analysis did not suggest any signs of liver damage as indicated by insignificant alteration of aspartate transaminase (AST), alanine transaminase (ALT), and glutamate dehydrogenase (GLDH) levels (Fig. 7A). However, significant decreases in magnesium, potassium, and triglycerides could indicate a poorer gastrointestinal absorption or altered kidney function (Fig. 7A). The hematologic analysis suggested no signs of inflammation, although a tendency toward reduced leukocytes and reticulocytes, as well as a moderate but significant reduction in erythrocytes and hemoglobin was noted, which may indicate moderately altered hematopoiesis (Fig. 7B). These alterations are possibly caused by changes in WNT/β-catenin and YAP signaling activities, as both pathways are known regulators of hematopoiesis (42, 43).

In conclusion, toxicity in mice at 100 mg/kg OM-153 oral–twice daily dosing is, at least in partially, caused by intestinal damage. In contrast, a 10 mg/kg OM-153 oral–twice daily dosing was overall tolerated in mice, and the intestinal architecture was intact despite a diminished *Lgr5* expression in ISCs at the crypt the base.

## Discussion

Here we show that OM-153 is a highly potent and specific inhibitor of TNKS1/2, WNT/β-catenin, YAP and MYC signaling capable of reducing cell growth in a subset of human cancer cell lines. Oral–twice daily dosing of 0.33–10 mg/kg OM-153 in COLO 320DM mouse colon carcinoma xenografts attenuated WNT/β-catenin signaling and resulted in a 74%–85% tumor growth inhibition. Moreover, in the isogenic and immunocompetent B16-F10 mouse melanoma model, oral–twice daily dosing of 0.1–10 mg/kg OM-153 potentiated efficacy of anti–PD-1 treatment and resulted in a 51%–65% tumor growth inhibition.

Deregulated WNT/β-catenin signaling contributes to aberrant cell growth and carcinogenesis, but is in parallel also critical for stem cell renewal, proliferation, and differentiation, during both embryogenesis and tissue homeostasis (20, 44). In the last decade, two studies using the early-generation TNKS1/2 inhibitors G007-LK and G-631, observed TNKSi-induced anti–colon carcinoma efficacies (33, 34). These reports also describe severe side-effects at therapeutic doses including intestinal toxicity limiting the therapeutic window (33, 34, 45).

In the 28-day repeated dose mouse toxicity study, when dosing mice oral–twice daily with 100 mg/kg OM-153, we indeed documented rapid loss of body weight, intestinal epithelial degeneration, and inflammation, as well as tubular damage in the kidney. However, we were not able to detect substantial side-effects and toxicity in mice treated with oral–twice daily dosing using 10 mg/kg, and importantly, their intestinal architecture was intact. In the ileum of these mice, MKI67 staining documented continual proliferation in the crypts and KRT20 staining showed differentiated epithelial cells. Similar to our previous report, showing TNKSi-induced elimination of differentiated cells traced from WNT/β-catenin signaling–dependent LGR5^+^ ISCs (41), *Lgr5* expression was lost and the result may propose the presence of a TNKSi-resistant ISC population. Hence, one may hypothesize that intestinal proliferation from dispensable LGR5^+^ ISCs (46) can be maintained by activated proliferation of an alternative source of stem cells. Quiescent, WNT/β-catenin signaling-independent, LGR5^−^/BMI1 proto-oncogene, polycomb ring finger (BMI1)^+^ and crypt +4-positioned ISCs have been described in that role earlier (41, 47, 48). Comprehensive follow-up studies of possible TNKSi-resistant ISC populations are evidently required.

We note that the primary pharmacologic on-target effect for OM-153 is seen in the low nanomolar range, as indicated by the TNKS1/2, WNT/β-catenin signaling reporter IC_50_-values, and COLO 320DM GI_50_ value, while the micromolar range *C*_max_ values for oral–twice daily dosing with 10 and 100 mg/kg OM-153 are more than 2 logs higher (5.3 and 39.2 μmol/L, respectively). The results propose that the documented toxicity is likely not coupled to on-target inhibition of TNKS1/2 and WNT/β-catenin signaling, but rather caused by concentration-dependent interactions with unknown off-targets, or optionally related to the physicochemical properties of OM-153 (45). Further detailed mechanistic investigation of plausible off-target toxicity is considered necessary. The possible compatibility of the tankyrase biotarget, with advancing tankyrase-specific inhibitors toward clinical trials in the cancer arena, has recently been demonstrated by the initiation of clinical trials with the TNKSi STP1002 (37).

The therapeutic index is normally measured as the ratio of the highest exposure to the drug that results in no toxicity (i.e., toxic dose in 50% of subjects, TD_50_) to the exposure that produces the desired effect (i.e., efficacious dose in 50% of subjects, ED_50_; ref. 45). The therapeutic window for a drug is commonly known as the dose range that can treat a disease effectively without having toxic effects. When using monotherapy treatment, we document no adverse toxicity in mice treated with oral–twice daily dosing using 10 mg/kg OM-153, and a significant antitumor efficacy in the COLO 320DM colon carcinoma xenograft model when dosing mice oral–twice daily with 0.33–10 mg/kg OM-153. Together, these data indicate a therapeutic window ranging from 0.33 mg/kg to at least 10 mg/kg.

In conclusion, in our experimental setting, we show that OM-153 exhibits antitumor efficacy and a viable therapeutic window in mouse models, which rationalize further preclinical evaluations to translate TNKS1/2 inhibition to a human therapeutic setting.

## Supplementary Material

Supplementary Figures S1-S8 Supplementary Figure S1. Chemical structures of selected tankyrase inhibitors. Supplementary Figure S2. OM-153 specifically inhibits TNKS1/2 and WNT/β-catenin signaling. Supplementary Figure S3. OM-153 specifically inhibits cell growth of an APC-mutated colon carcinoma cell line. Supplementary Figure S4. OM-153 shows an anti-proliferative effect in human cancer cell lines. Supplementary Figure S5. OM-153 inhibits WNT/β-catenin, YAP and MYC signaling in human cancer cell lines. Supplementary Figure S6. OM-153 inhibits the WNT/β-catenin signaling pathway and shows anti-tumor effect in a human colon carcinoma xenograft model. Supplementary Figure S7. Combined OM-153 and anti-PD-1 treatment confers anti-tumor effect in mouse melanoma. Supplementary Figure S8. PO-BID treatment with 10 mg/kg OM-153 does not reduce body weight and food consumption or induce toxicity in mice.
