# Supplementary material for: The Tankyrase Inhibitor OM-153 Demonstrates Antitumor Efficacy and a Therapeutic Window in Mouse Models
Source: Cancer Res Commun. 2022 Apr 20;2(4):233–45. doi: 10.1158/2767-9764.CRC-22-0027 (PMC9981206; doi:10.1158/2767-9764.CRC-22-0027)
Supplement: Supplementary Figures S1-S8 — Supplementary Figure S1. Chemical structures of selected tankyrase inhibitors. Supplementary Figure S2. OM-153 specifically inhibits TNKS1/2 and WNT/β-catenin signaling. Supplementary Figure S3. OM-153 specifically inhibits cell growth of an APC-mutated colon carcinoma cell line. Supplementary Figure S4. OM-153 shows an anti-proliferative effect in human cancer cell lines. Supplementary Figure S5. OM-153 inhibits WNT/β-catenin, YAP and MYC signaling in human cancer cell lines. Supplementary Figure S6. OM-153 inhibits the WNT/β-catenin signaling pathway and shows anti-tumor effect in a human colon carcinoma xenograft model. Supplementary Figure S7. Combined OM-153 and anti-PD-1 treatment confers anti-tumor effect in mouse melanoma. Supplementary Figure S8. PO-BID treatment with 10 mg/kg OM-153 does not reduce body weight and food consumption or induce toxicity in mice. [file crc-22-0027-s01.pdf]

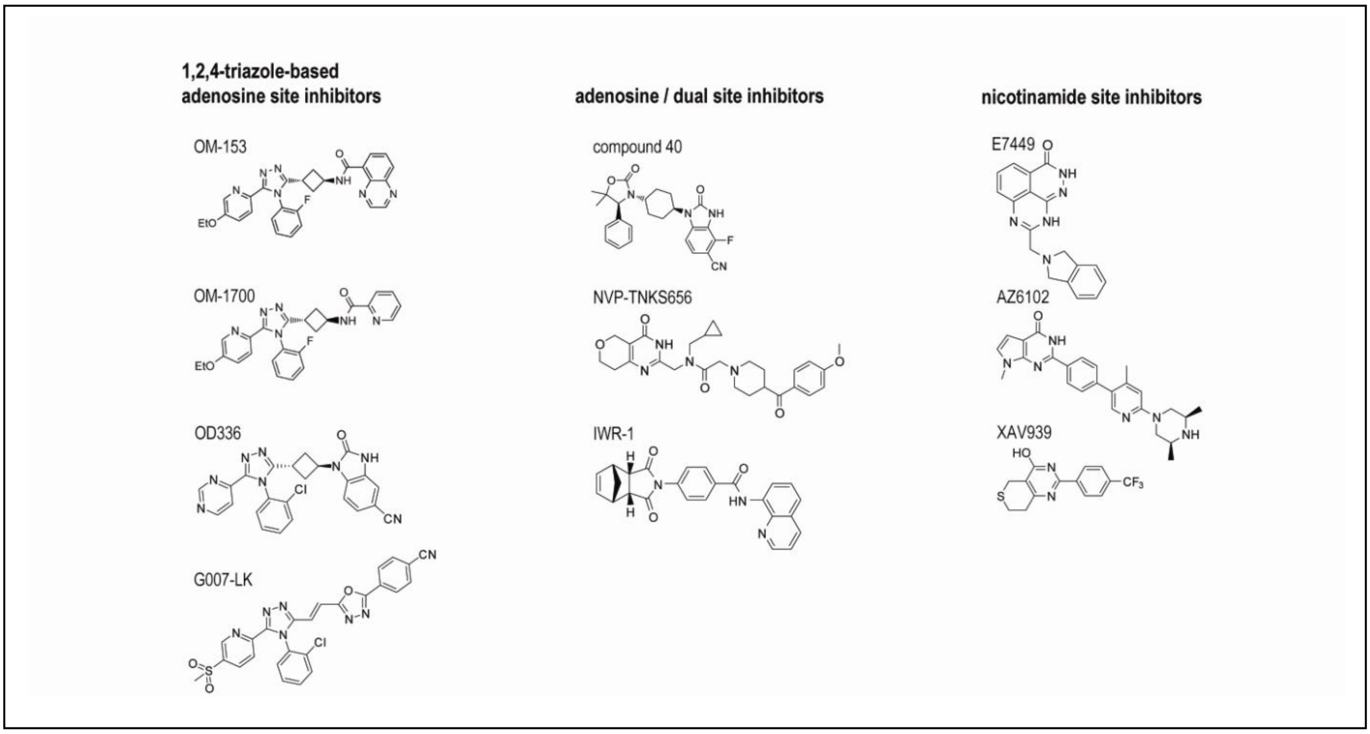

Supplementary Figure S1. Chemical structures of selected tankyrase inhibitors.

The compounds inhibit TNKS1/2 by binding to either TNKS adenosine or nicotinamide binding sites, or both (dual binder, NVP-TNKS565).

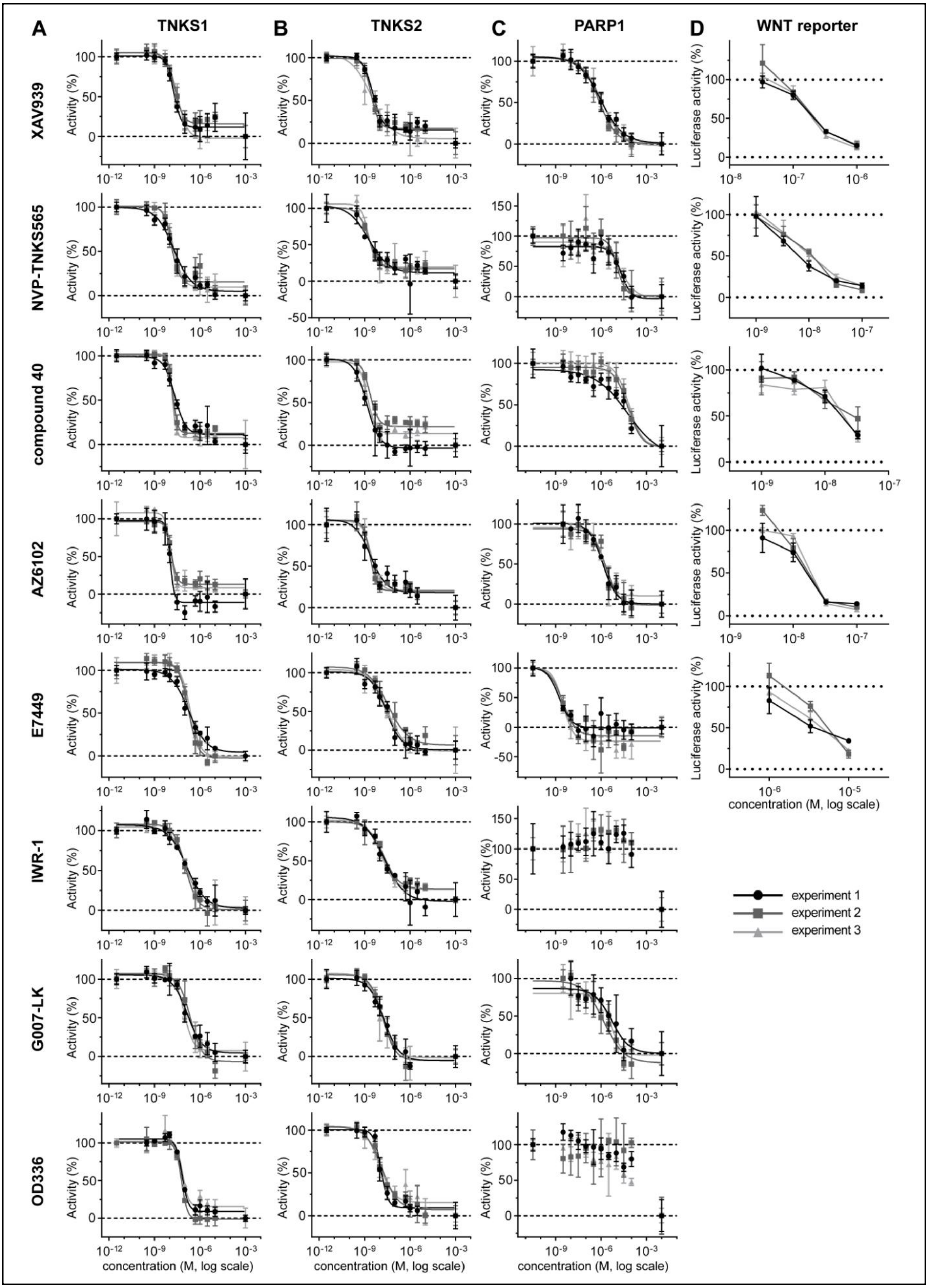
A
TNKS1
B
TNKS2
C
PARP1
D
WNT reporter
XAV939
NVP-TNKS565
compound 40
AZ6102
E7449
IWR-1
G007-LK
OD336
Activity (%)
Luciferase activity (%)
concentration (M, log scale)
experiment 1
experiment 2
experiment 3

Supplementary Figure S2. OM-153 specifically inhibits TNKS1/2 and WNT/β-catenin signaling.

IC_{50}-value determination experiments for inhibition of (**A**) TNKS1, (**B**) TNKS2 and (**C**) PARP1 (activity [%] in biochemical assays), and (**D**) luciferase-based WNT/β-catenin signaling reporter assay in HEK293 cells (relative and normalized luciferase activity [%], WNT reporter) by the depicted TNKS inhibitors. For the biochemical assays, datapoints from controls containing no enzyme were placed at concentrations 10^{-3} M (TNKS1/2) or 10^{-2} M (PARP1). Datapoints from controls containing no inhibitor were placed at concentrations $10^{-11.5}$ M (TNKS1/2) or $10^{-10.5}$ M (PARP1). Mean ± SD values for three independent experiments are shown.

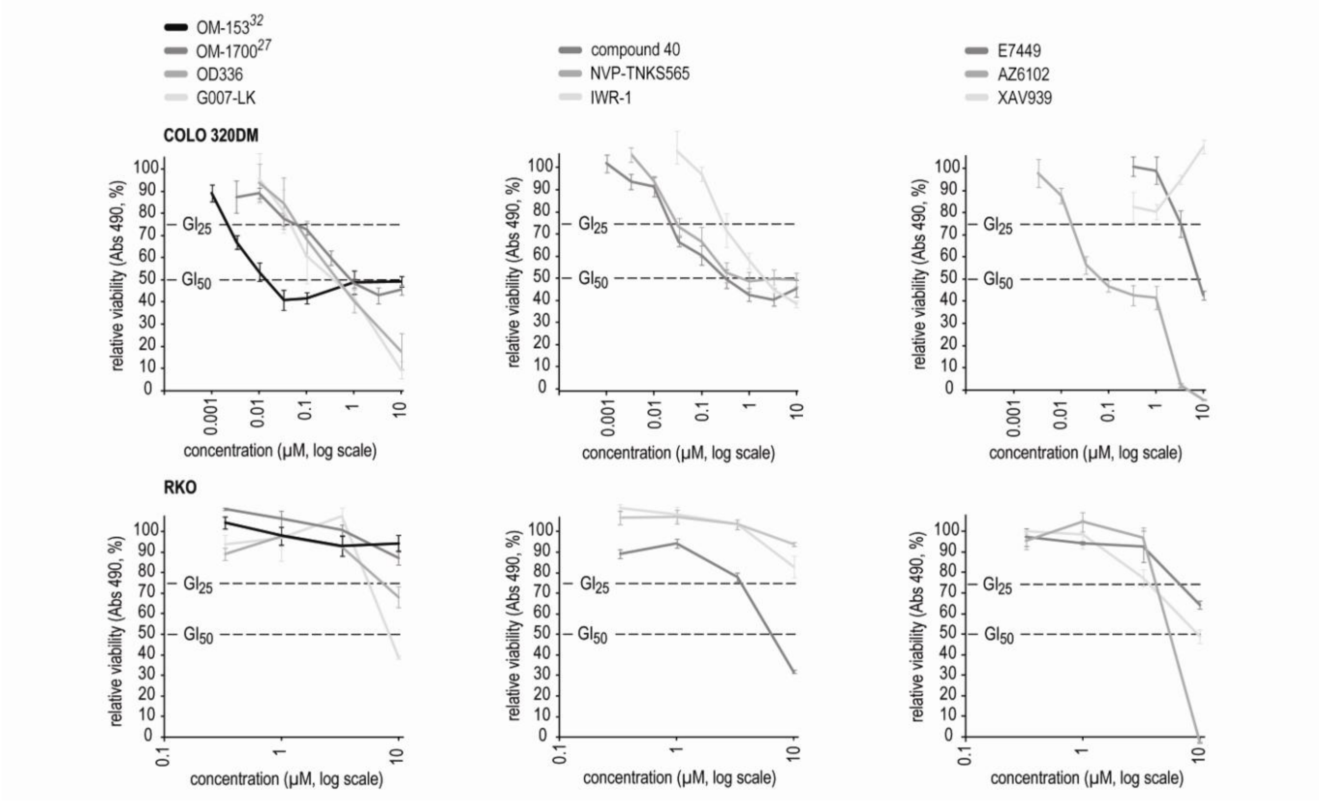

Supplementary Figure S3. OM-153 specifically inhibits cell growth of an *APC*-mutated colon carcinoma cell line.

MTS colorimetric cell growth assay (Abs 490) for various concentrations of tankyrase inhibitors in treated COLO 320DM (*APC*^{mutated} and WNT/β-catenin signaling-dependent, top panels) and RKO colon cancer cells (*APC*$^{wild\text{-}type}$ and WNT/β-catenin signaling-independent, bottom panels). GI_{50}- and GI_{25}-values (nM) were calculated relative to control (100%, 0.01% DMSO) and experiment time 0 values (Abs 490 t_0, set to 0%) after 5 days of cultivation. Numbers in superscript indicate data from reference 27 and 32. Mean ± SD values for data from one representative experiment of more than three repeated assays, each with six replicates, are shown.

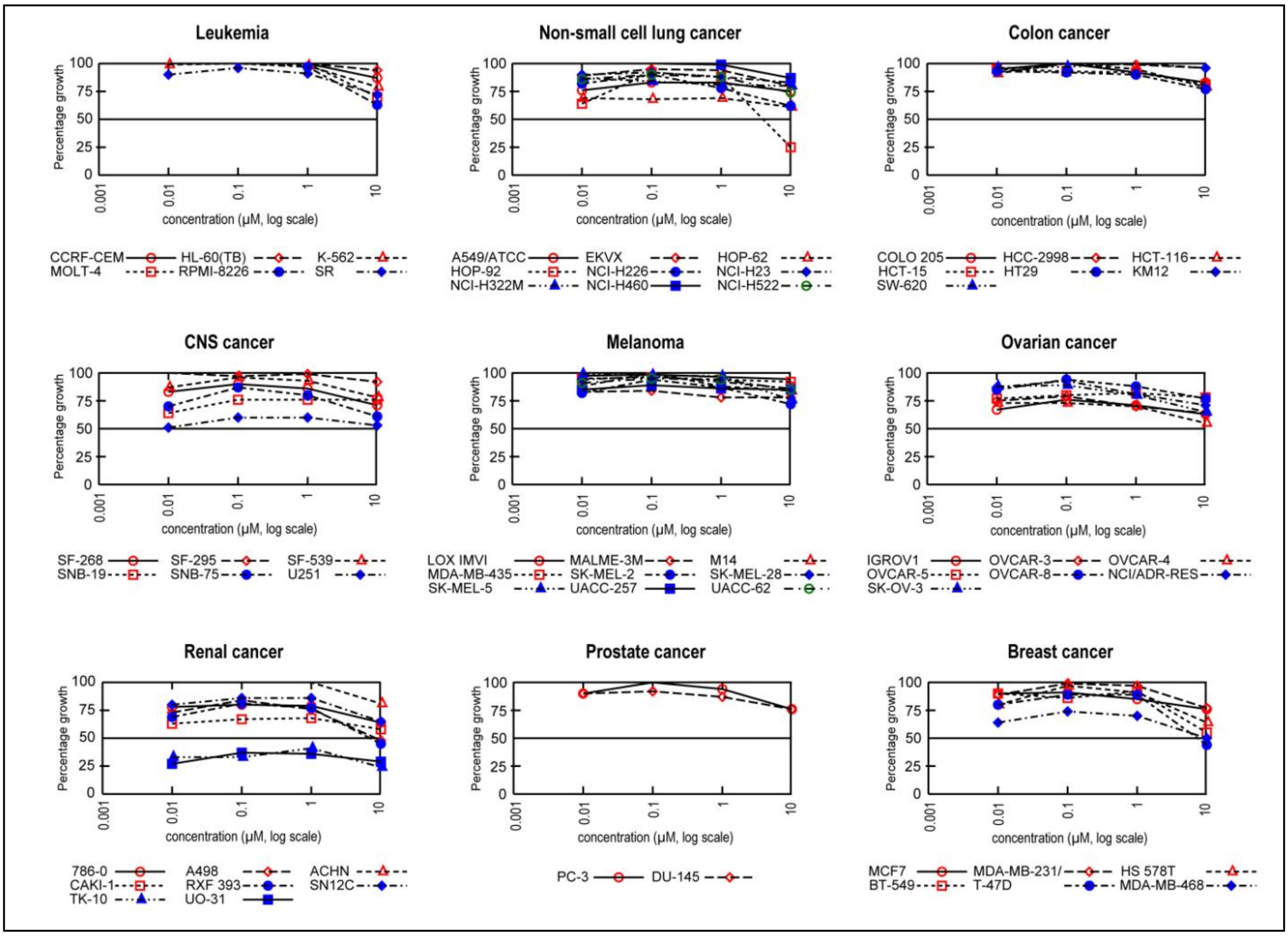

Supplementary Figure S4. OM-153 shows an anti-proliferative effect in human cancer cell lines.

NCI-60 human cancer cell line proliferation/viability screen using indicated concentrations of OM-153 for 48 hours. Relative percentage growth was calculated relative to control (100%, 0.01% DMSO) and experiment time 0 values (t_0, set to 0%).

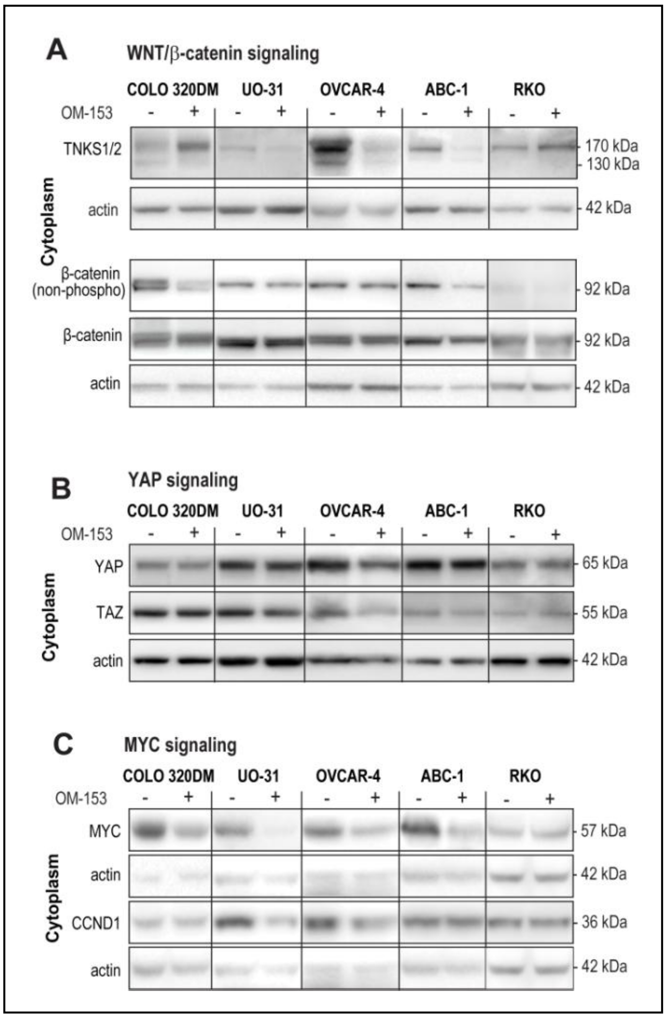

Supplementary Figure S5. OM-153 inhibits WNT/β-catenin, YAP and MYC signaling in human cancer cell lines.

(**A**) Evaluation of WNT/β-catenin signaling components. Immunoblots of cytoplasmic TNKS1/2, the active form of β-catenin (non-phosho [Ser33/37/Thr41]) and total β-catenin.

For **A-C**, cells were treated for 24 hours with OM-153 (10 nM) or controls (0.0001% DMSO). Immunoblots show representative data from two or more independent experiments. Actin was used as loading controls.

(**B**) Evaluation of YAP signaling. Immunoblots of cytoplasmic YAP and TAZ.

(**C**) Evaluation of MYC signaling. Immunoblots of cytoplasmic MYC and CCND1.

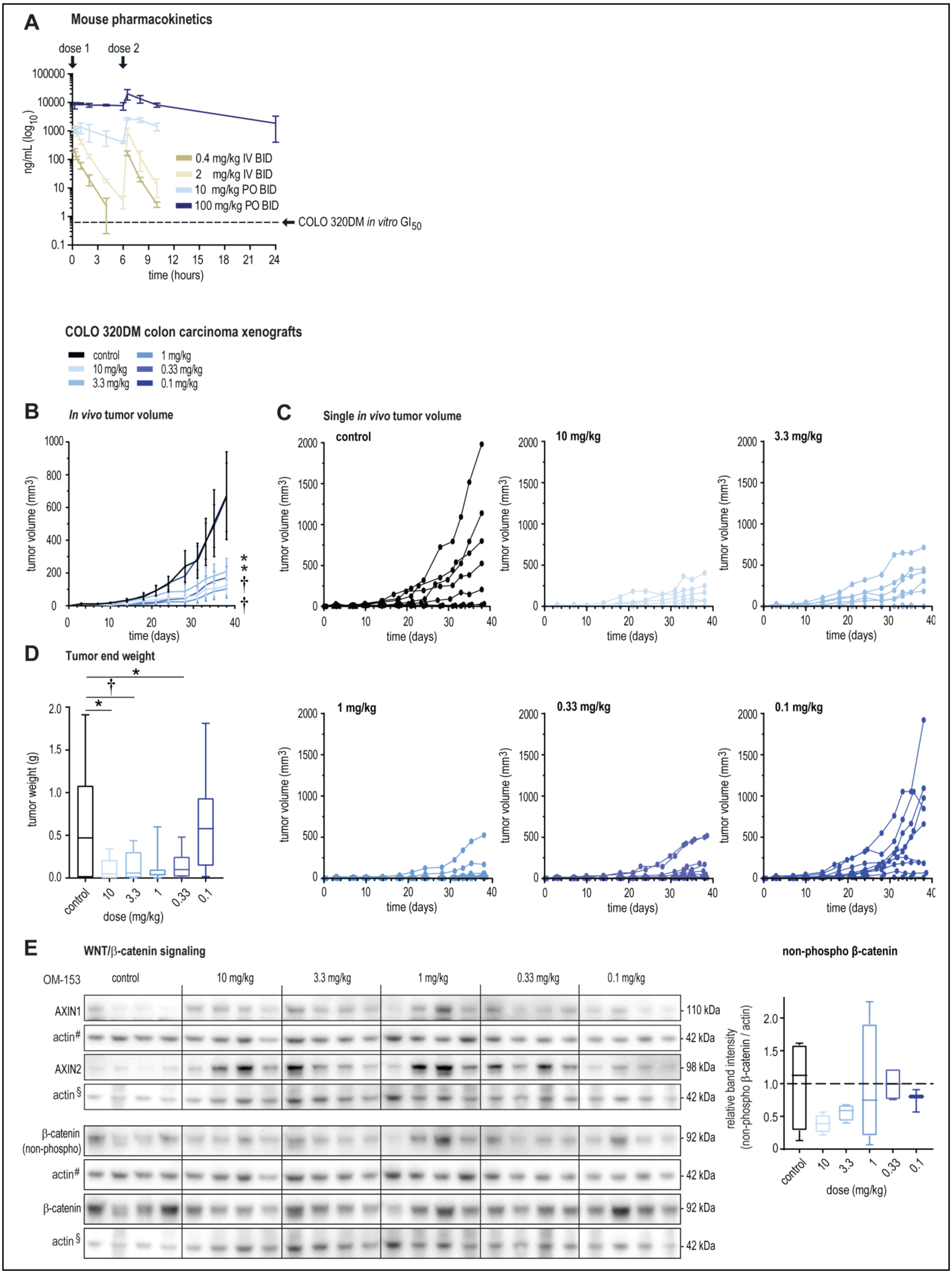
A
Mouse pharmacokinetics
dose 1
dose 2
ng/mL (log10)
0.4 mg/kg IV BID
2 mg/kg IV BID
10 mg/kg PO BID
100 mg/kg PO BID
COLO 320DM in vitro GI50
time (hours)
COLO 320DM colon carcinoma xenografts
control
10 mg/kg
3.3 mg/kg
1 mg/kg
0.33 mg/kg
0.1 mg/kg
B
In vivo tumor volume
tumor volume (mm3)
time (days)
C
Single in vivo tumor volume
D
Tumor end weight
tumor weight (g)
dose (mg/kg)
E
WNT/β-catenin signaling
OM-153
AXIN1
actin#
AXIN2
actin§
β-catenin (non-phospho)
β-catenin
110 kDa
42 kDa
98 kDa
92 kDa
non-phospho β-catenin
relative band intensity (non-phospho β-catenin / actin)

Supplementary Figure S6. OM-153 inhibits the WNT/β-catenin signaling pathway and shows anti-tumor effect in a human colon carcinoma xenograft model.

(**A**) Mouse pharmacokinetics upon intravenous (i.v., 0.4 or 2 mg/kg) or oral (p.o., 10 or 100 mg/kg) administration of OM-153 either once (group 1) or twice-daily (BID) in CD-1 mice (group 2). COLO 320DM *in vitro* GI_{50}-value (10 nM = 5.1 ng/mL) is depicted by stippled line.

(**B**) *In vivo* tumor volume (mm^3, mean ± SD values are shown). One-tailed t-tests, * ($P < 0.05$) for 3.3 and 0.33 mg/kg OM-153, and one-tailed Mann-Whitney rank sum test, † ($P < 0.05$) for 10 and 0.1 mg/kg OM-153, all versus vehicle control.

For **B**-**E**, from COLO 320DM-challenged (subcutaneous, s.c.) CB17-SCID mice upon treatment with 10 (n = 8), 3.3 (n = 10), 1 (n = 9), 0.33 (n = 9) or 0.1 (n = 9) mg/kg OM-153 and vehicle control (n = 7) from day 4 through 37 (all treated oral-twice daily, p.o. BID).

(**C**) Single i*n vivo* tumor volume (mm^3).

(**D**) Tumor end weight (right panel). Boxplots show median, first and third quartiles and maximum and minimum whiskers. One-tailed t-tests, * ($P < 0.05$), and one-tailed Mann-Whitney rank sum tests, † ($P < 0.05$), all versus vehicle control.

(**E**) Representative immunoblots showing altered expression of AXIN1, AXIN2, the active form of β-catenin (non-phosho [Ser33/37/Thr41]) and β-catenin (total), left panel. Quantified protein immunoblot ratios (protein vs. loading control) for non-phosho β-catenin, right panel. n = 4 tumors, collected 4 hours post last dosing, were analyzed for each treatment group and stippled lines depict control mean values = 1. § and # indicates use of duplicated loading controls (actin).

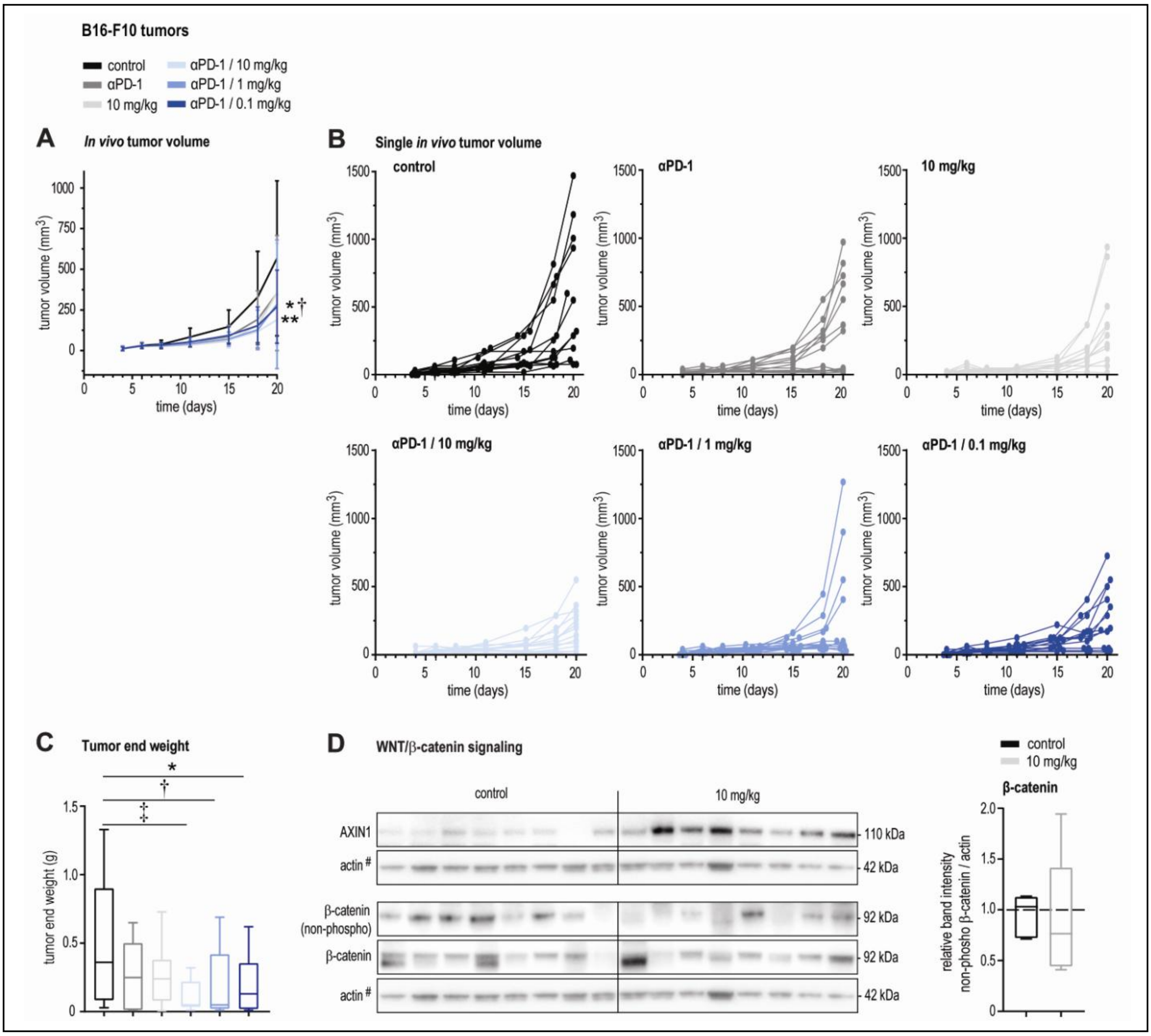
B16-F10 tumors
control
αPD-1
10 mg/kg
αPD-1 / 10 mg/kg
αPD-1 / 1 mg/kg
αPD-1 / 0.1 mg/kg
A
In vivo tumor volume
tumor volume (mm3)
time (days)
B
Single in vivo tumor volume
control
αPD-1
10 mg/kg
αPD-1 / 10 mg/kg
αPD-1 / 1 mg/kg
αPD-1 / 0.1 mg/kg
C
Tumor end weight
tumor end weight (g)
D
WNT/β-catenin signaling
control
10 mg/kg
AXIN1
actin #
β-catenin (non-phospho)
β-catenin
110 kDa
42 kDa
92 kDa
β-catenin
relative band intensity non-phosho β-catenin / actin

Supplementary Figure S7. Combined OM-153 and anti-PD-1 treatment confers anti-tumor effect in mouse melanoma.

(**A**) *In vivo* tumor volume (mm^3, mean ± SD values are shown). One-tailed t-tests, ** ($P < 0.001$) for αPD-1/10 mg/kg OM-153 and * ($P < 0.05$) for αPD-1/0.1 mg/kg OM-153. One-tailed Mann-Whitney rank sum test, † ($P < 0.05$) for αPD-1/1 mg/kg OM-153. All versus vehicle control.

For **A-C**, from B16-F10-challenged (s.c.) C57BL/6N mice upon treatment from day 6 through 20. Boxplots show median, first and third quartiles and maximum and minimum whiskers. Vehicle control (n = 12), anti-(α)PD-1 (n = 13), 10 mg/kg OM-153 (n = 11), and combined treatment with αPD-1 and 10 (n = 13), 1 (n = 13) and 0.1 (n = 13) mg/kg OM-153. All OM-153 treatments, oral-twice daily.

(**B**) Single i*n vivo* tumor volume (mm^3).

(**C**) Tumor end weight (right panel). Boxplots show median, first and third quartiles and maximum and minimum whiskers. One-tailed t-tests, * ($P < 0.05$), and one-tailed Mann-Whitney rank sum tests, ‡ ($P < 0.01$) and † ($P < 0.05$), all versus vehicle control.

(**D**) Representative immunoblots showing altered expression of AXIN1, the active form of β-catenin (non-phosho [Ser33/37/Thr41]) and total β-catenin upon treatment with vehicle control or 10 mg/kg OM-153 (both n = 8, collected 8 hours post last dosing). # indicates use of duplicated loading controls (actin), left panel. Quantified protein immunoblot ratios (protein vs. loading control) for total β-catenin and stippled line depicts control mean value = 1, right panel.

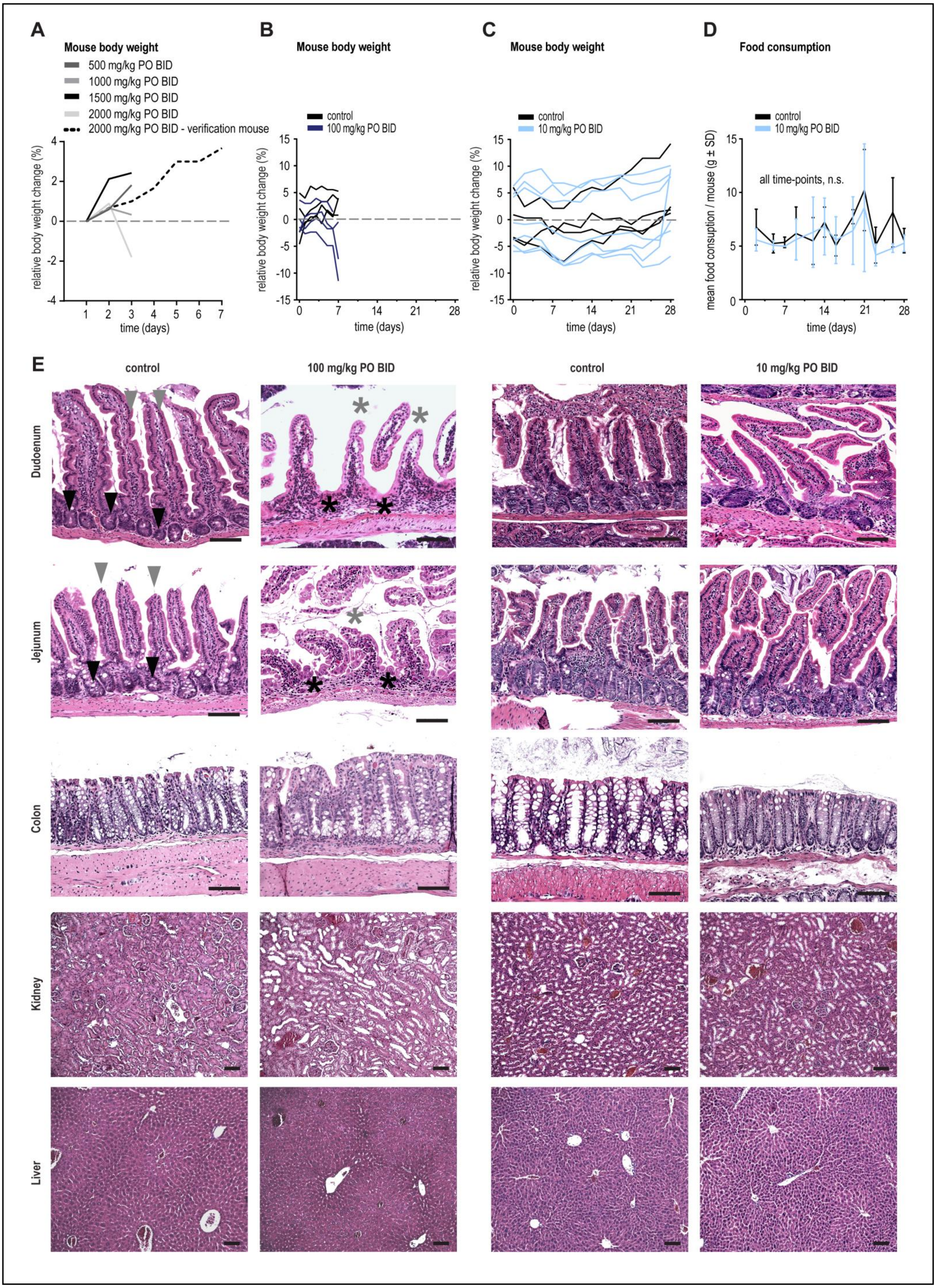

A
Mouse body weight
500 mg/kg PO BID
1000 mg/kg PO BID
1500 mg/kg PO BID
2000 mg/kg PO BID
2000 mg/kg PO BID - verification mouse
relative body weight change (%)
time (days)
B
Mouse body weight
control
100 mg/kg PO BID
relative body weight change (%)
time (days)
C
Mouse body weight
control
10 mg/kg PO BID
relative body weight change (%)
time (days)
D
Food consumption
control
10 mg/kg PO BID
all time-points, n.s.
mean food consuption / mouse (g ± SD)
time (days)
E
control
100 mg/kg PO BID
control
10 mg/kg PO BID
Dudoenum
Jejunum
Colon
Kidney
Liver

Supplementary Figure S8. Oral-twice daily treatment with 10 mg/kg OM-153 does not reduce body weight and food consumption or induce toxicity in mice.

(**A**) Relative individual body weights in CD-1 mice noted through three days upon single treatment with 500, 1000, 1500 or 2000 mg/kg OM-153 (n = 1), or through seven days upon single treatment with 2000 mg/kg OM-153 (verification mouse, n = 1).

For **A**-**C**, mean body weight at experiment initiation is set at 0%.

For **A**-**E**, all treatments, oral-twice daily (p.o, BID)

(**B**) Relative individual body weights upon 7 days of treatment with 100 mg/kg OM-153 (n = 4) and vehicle control (n = 4) in C57BL/6J mice.

(**C**) Relative individual body weights upon 28 days of treatment with 10 mg/kg OM-153 (n = 7) and vehicle control (n = 4) in CD-1 mice.

(**D**) Food consumption (g) per mouse, day and cage treated with 10 mg/kg OM-153 (n = 3 cages) and vehicle control (n = 2 cages) as described in **A**. Means ± SDs are shown and n.s. = not significant.

(**E**) Representative pictures taken from multiple sections of H&E-stained duodenum, jejunum, colon, kidney and liver from mice treated for 28 days with with 100 mg/kg OM-153 (n = 4) and vehicle control (n = 4) in C57BL/6J mice (left panels), or 10 mg/kg OM-153 (n = 7) and vehicle control (n = 4) in CD-1 mice (right panels). Signs of acute tubular damage in the kidney of 100 mg/kg-treated animals included vacuolization of tubular epithelium, dilated tubuli with flattened epithelium. Scale bars: 200 µm (original magnification ×200) for duodenum, jejunum and colon, and 100 µm (original magnification ×100) for kidney and liver. Arrowheads highlight intact intestinal villi (grey) and crypts (black), while asterixes highlight blunted villi (grey) and lost crypts (black).
